# Smart Watch Versus Classic Receivers: Static Validity of Three GPS Devices in Different Types of Built Environments

**DOI:** 10.3390/s21217232

**Published:** 2021-10-30

**Authors:** Michal Vorlíček, Tom Stewart, Jasper Schipperijn, Jaroslav Burian, Lukáš Rubín, Jan Dygrýn, Josef Mitáš, Scott Duncan

**Affiliations:** 1 Faculty of Physical Culture, Palacký University Olomouc, 771 11 Olomouc, Czech Republic or lukas.rubin@tul.cz (L.R.); jan.dygryn@upol.cz (J.D.); josef.mitas@upol.cz (J.M.); 2 School of Sport and Recreation, Auckland University of Technology, Auckland 1010, New Zealand; tom.stewart@aut.ac.nz (T.S.); scott.duncan@aut.ac.nz (S.D.); 3 Department of Sport Science and Clinical Biomechanics, University of Southern Denmark, 5230 Odense, Denmark; jschipperijn@health.sdu.dk; 4 Faculty of Science, Palacký University Olomouc, 771 11 Olomouc, Czech Republic; jaroslav.burian@upol.cz; 5 Faculty of Science, Humanities and Education, Technical University of Liberec, 461 17 Liberec, Czech Republic

**Keywords:** accuracy, environment, GPS, logger, geodetic point, Garmin smart watch

## Abstract

In order to study the relationship between human physical activity and the design of the built environment, it is important to measure the location of human movement accurately. In this study, we compared an inexpensive GPS receiver (Holux RCV-3000) and a frequently used Garmin Forerunner 35 smart watch, with a device that has been validated and recommended for physical activity research (Qstarz BT-Q1000XT). These instruments were placed on six geodetic points, which represented a range of different environments (e.g., residential, open space, park). The coordinates recorded by each device were compared with the known coordinates of the geodetic points. There were no differences in accuracy among the three devices when averaged across the six sites. However, the Garmin was more accurate in the city center and the Holux was more accurate in the park and housing estate areas compared to the other devices. We consider the location accuracy of the Holux and the Garmin to be comparable to that of the Qstarz. Therefore, we consider these devices to be suitable instruments for locating physical activity. Researchers must also consider other differences among these devices (such as battery life) when determining if they are suitable for their research studies.

## 1. Introduction

Over the past 15 years, there has been an increase in scientific publications focusing on the assessment of the environmental correlates of physical activity (PA) [1,2,3,4]. One of the reasons for this increase is the rapid evolution of modern technologies, including the more accurate measurement of PA levels as well as advances in geographic information systems (GIS) and related technologies. There has also been a significant shift in geospatial positioning technology, most notably with the Global Navigation Satellite System (GNSS), which joins the European Galileo system and the Russian GLONASS system with the Global Positioning System (GPS). These technologies, which are used in conjunction with kinanthropology research, have led to an enhanced understanding of the complex relationships between human movement behavior and the built environment [5,6,7]

The spatial location of PA has been measured across all population groups [8,9], with GPS data most commonly combined with objective measurements of PA using accelerometers [10,11,12]. GPS data can also be used to more accurately specify the type of PA, such as walking, running, cycling, or traveling in a vehicle [13,14,15,16]. It can also be used to detect time spent indoors [17]. Satellite technologies can also be used to identify environments associated with risky behaviors such as smoking, drinking, and using drugs. It is very important to consider this type of information is in order to avoid the negative impacts of urban development while promoting active living in the population [18].

GPS receivers are the most commonly used measurement instruments in current research on geospatial information associated with PA [19,20,21,22]. When using GPS devices in scientific research, it is necessary to use instruments that provide accurate data. One way to verify the accuracy of a GPS instrument is through static validity, where the instrument is placed on a predefined geodetic point in a selected environment. The recorded coordinates are then compared to the known coordinates of the geodetic point [23].

To date, numerous validation studies focusing on the validity of GPS receivers in team sports [24,25,26] have been carried out, but only a limited number of studies have investigated the validity of GPS receivers in free-living conditions [27]. Moreover, these studies mainly focus on one type of device, such as smartphones [28,29,30], and on one specific environment or location [31,32,33]. According to previous studies [34,35,36,37], the accuracy of this information can vary significantly in different environments, so it is important to test these devices across several environmental settings. As these devices can be reasonably expensive, particularly in studies with a large number of participants, it is also important to test the accuracy of less expensive devices and non-research-grade consumer wearables (e.g., smart watches). This information can help researchers to choose the most appropriate device for their studies.

Therefore, the purpose of this study is to compare the static accuracy of (1) an inexpensive and unverified GPS receiver (Holux RCV-3000), (2) one of the most widely used [38] smart watches (Garmin Forerunner 35), and (3) the research-validated and expert-recommended [15,23] Qstarz BT-Q1000XT GPS receiver in diverse environmental conditions. We hypothesize that the cheaper GPS receiver (Holux) and the smart watch (Garmin) will not be as accurate as the Qstarz device.

## 2. Materials and Methods

This section describes how the data were collected and provides details of the devices used. The data processing steps and statistical analysis procedures are then described.

### 2.1. Procedure

**Figure 1:**
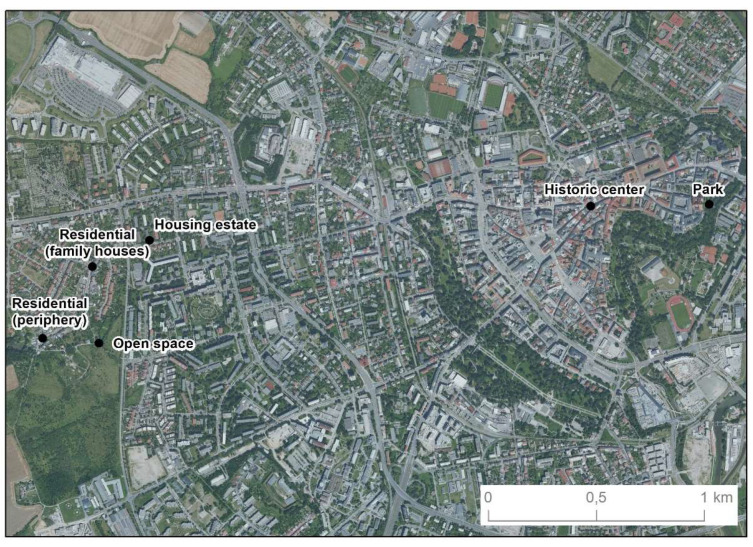
The location of the six selected geodetic points.

**Figure 2:**
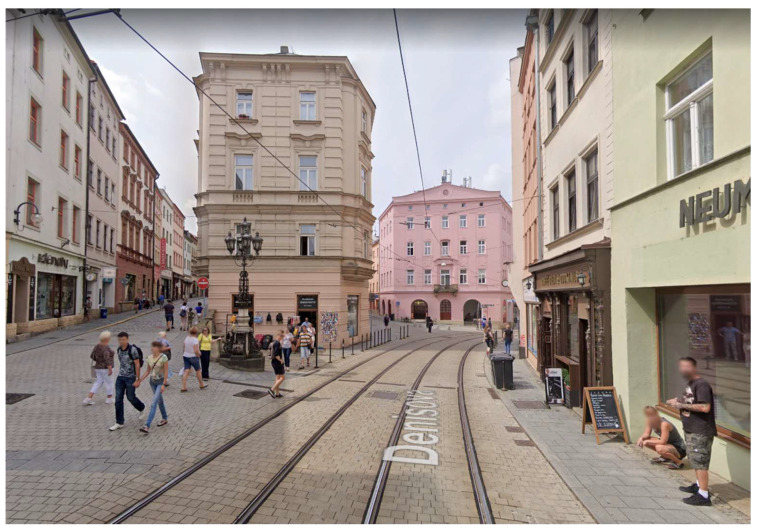
The historic center environment.

**Figure 3:**
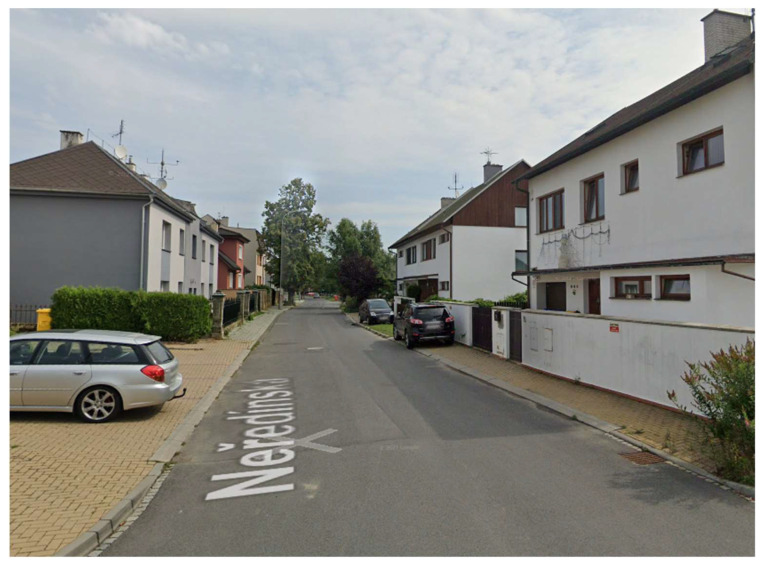
The residential (family houses) environment.

**Figure 4:**
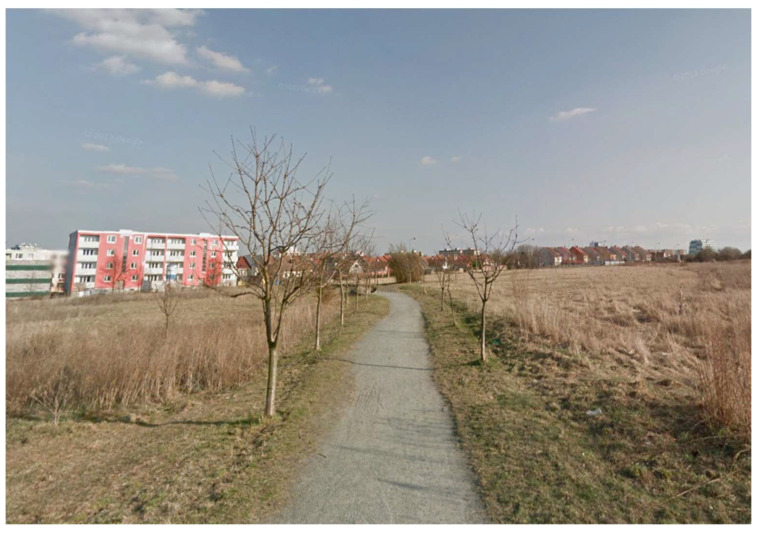
The open space environment.

**Figure 5:**
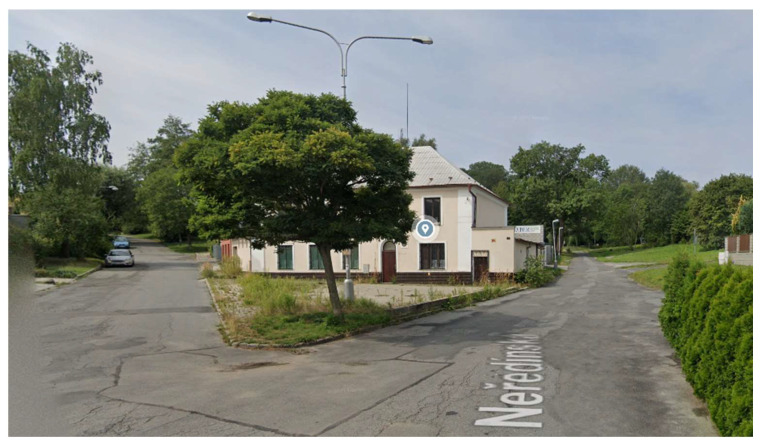
The residential (periphery) environment.

**Figure 6:**
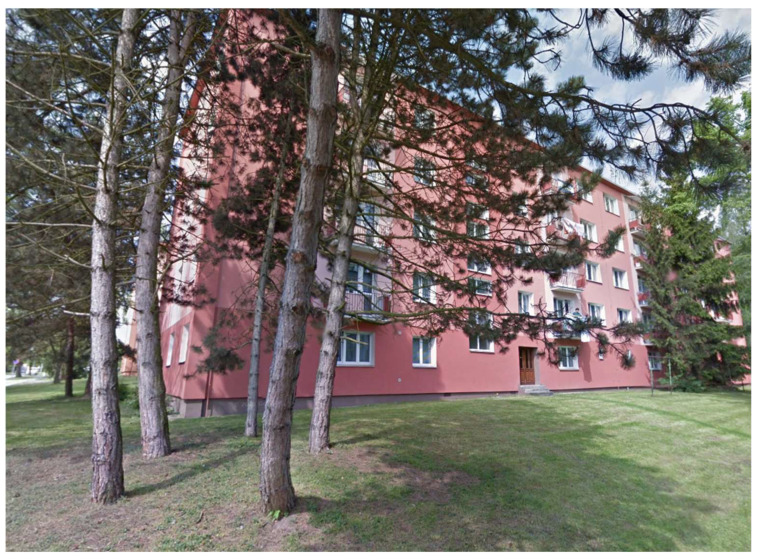
The housing estate environment.

**Figure 7:**
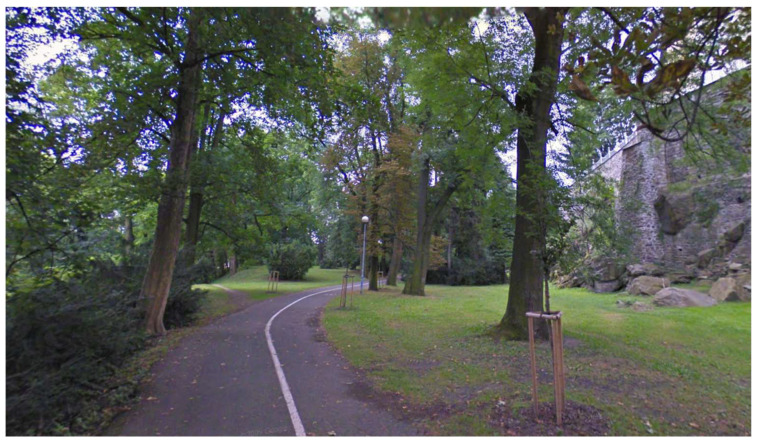
The park environment.

Spatial data were recorded at six selected geodetic points (Figure 1) in 2018 using the Holux RCV-3000 and Qstarz BT-Q1000XT GPS receivers and the Garmin Forerunner 35 smart watch. Geodetic points were selected across different types of built environment (historic center (Figure 2), residential (family houses) (Figure 3), open space (Figure 4), residential (periphery) (Figure 5), housing estate (Figure 6) and park (Figure 7)) in the city of Olomouc (49°35′ N, 17°15′ E, 219 m above sea level) in the Czech Republic.

At each outside geodetic point, two instruments of each type were placed on a pad on the geodetic point for 60 min. The position recording interval for all three devices was set to one second (1 Hz sampling frequency). The main characteristics of the different types of built environments are as follows: historic center—historic buildings, narrow streets, multi-story houses (usually 4 floors), no vegetation cover, poor sky visibility; residential (family houses)—single-family houses, mostly multi-story (usually 2 floors) that line the roadway with sidewalks, vegetation in the form of hedges, good sky visibility; open space—gravel road on the edge of the city, no vegetation cover, no buildings close by, excellent sky visibility; residential (periphery)—isolated family houses, mostly multi-story (usually 2 floors), lower housing density, roadway without pavements, generally less compact development with more green space (e.g., gardens), vegetation also in the form of hedges and trees, good sky visibility; housing estate—multi-story blocks of flats (usually 5 floors) with the usual spacing between buildings, complemented by tall coniferous and deciduous trees, good sky visibility; park—park in historic center, close to medieval city walls (height approx. 5 m), large number of tall deciduous trees, poor sky visibility.

### 2.2. Devices

#### 2.2.1. Holux RCV-3000

**Figure 8:**
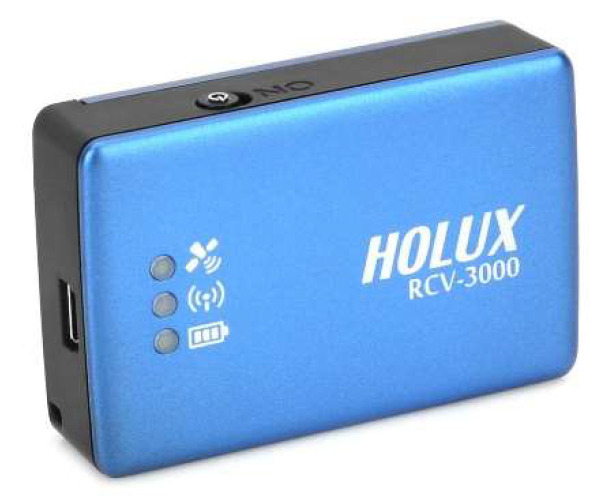
GPS receiver Holux RCV-3000.

This GPS receiver (Figure 8) contains a highly sensitive MediaTek MT3329 chip with parallel signal search on 66 channels and up to 22 tracking channels for fast positioning and recovery after GPS signal loss. A built-in WAAS/EGNOS demodulator ensures precise operation in dense buildings, valleys, and other environments with poor signal reception. The 4 MB internal memory can record up to 200,000 readings, logged by time or distance. The manufacturer states an accuracy of <3 m circular error probability (CEP) without SA. If EGNOS/WAAS are enabled, it is <2.2 m (horizontal, 95% of the time) and <5 m (vertical, 95% of the time). Routes can be tracked in Google Earth’ or with the included ezTour software. With support for the NMEA 0183 v3.01 data protocol, the Holux RCV-3000 can be used with all current GPS applications, navigation software, and mapping and tracking programs. The module can be used for hiking as well as in transport vehicles, including ships and aircraft. The module is also designed for use in extreme conditions (from −10 ℃ to +60 ℃) and includes protection from overheating and short circuiting. The weight of the device is 53 g and the size is 62.5 × 41 × 17.1 mm [39].

#### 2.2.2. Qstarz BT-Q1000XT

**Figure 9:**
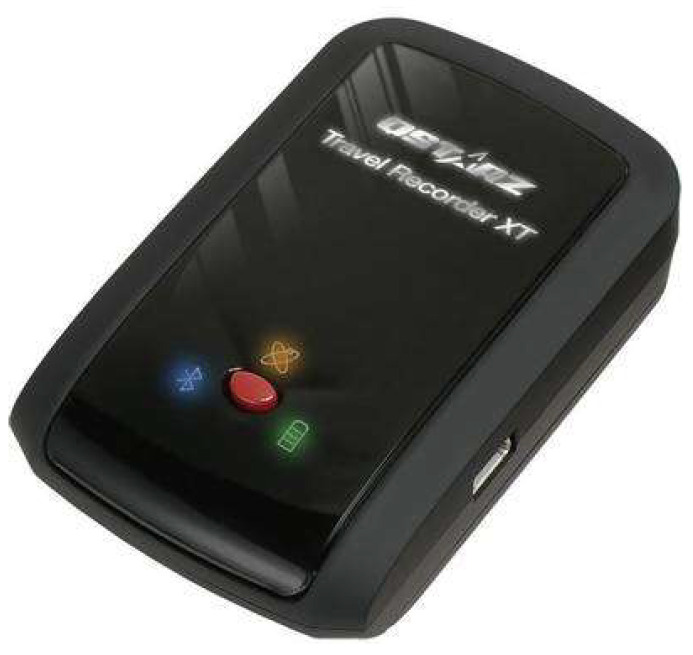
GPS receiver Qstarz BT-Q1000XT.

The receiver (Figure 9) contains an MTK II GPS chip (−165 dBm sensitivity) with 66 channels. It supports differential GPS augmentation systems (WAAS, EGNOS, and MSAS). Like the Holux, it is compliant with the NMEA 0183 (v3.01) data specification and has an operating temperature range of −10 ℃ to +60 ℃. The manufacturer’s specified accuracy is <3 m CEP (50%) without SA (horizontal) DGPS (WAAS, ENGOS, MSAS) 2.5 m. It is 72.2 × 46.5 × 20 mm in size and weighs 64.7 g.

#### 2.2.3. Garmin Forerunner 35

**Figure 10:**
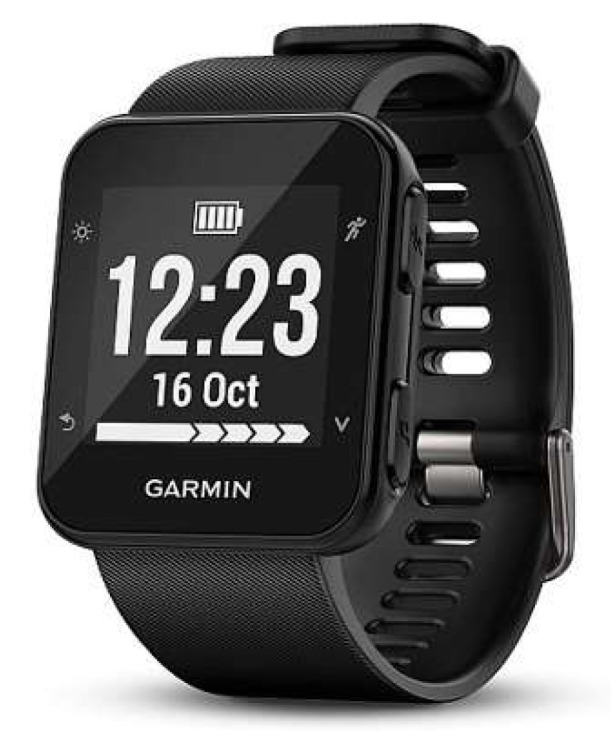
Smart watch Garmin Forerunner 35.

The Garmin Forerunner 35 (Figure 10) is one of Garmin’s widely used smart watches. It was launched on the market in 2016, and with a price of ~$200 USD (Table 1) it was a mid-range model (one of the cheapest models equipped with a GPS receiver). Unfortunately, the watch does not allow receiving signals from other GNSS networks (GLONASS or Galileo). the watch is equipped with an accelerometer to monitor indoor movement activity. As of 2021, this type has been replaced by newer models; however, its use remains widespread among the public.

### 2.3. Data Processing

The deviation between the position recorded by the GPS device and the coordinates of each geodetic point was calculated using the Haversine equation [40]. This approach is most commonly used to calculate the shortest spherical distance between two points. The geographic information system Esri ArcGIS for Desktop 10.6.1 was used for the visual interpretation of the data. Circular error probability (CEP), which is a measure of the horizontal positioning accuracy used in navigation, was also calculated. The probable circular error value defines the radius of a circle centered at a given point within which the correct horizontal position of the waypoint is likely to be found 50% of the time. In our case, the center of the circle was a given geodetic point and the radius was set so that 50% of the points recorded by each instrument were inside the circle.

### 2.4. Statistical Analysis

Descriptive statistics (mean, SD, median) were calculated to summarize the average deviation (error) from the geodetic points for each device (Holux, Qstarz, and Garmin), for each of the six sites (historic center, residential (family houses), residential (periphery), open space, housing estate, and park). Linear mixed models were used to determine if the error varied among the three devices and across the sites. The deviation from the geodetic point (m) was the dependent variable, while device (three levels), site (six levels), and the interaction between device and site were specified as fixed effects. The unique device ID (six devices; two of each model) was specified as a random effect to account for the within-device repeated measures. Estimated means and pairwise contrasts between each device and site were calculated, with multiple comparisons adjusted using the Bonferroni correction. Lastly, the Qstarz and Holux data were then combined and compared against the Garmin data, to test for any difference between commercial GPS receivers and consumer wearables. All mixed models were fit using the lme4 R package, and the estimation of means and contrasts was performed using the emmeans R package. The level of statistical significance was set at *p* < 0.05, and all analyses were performed in R version 4.1.1.

## 3. Results

A total of 131,543 measured GPS points were analyzed. On average 21,924 ± 249 GPS points were recorded at each geodetic point. Each instrument stored an average of 21,924 ± 168 points over the six sites. The total number of measured points was very evenly distributed (Table 2) among all six instruments and geodetic points. The greatest number of points were recorded at the housing estate (22,131) and the fewest in the historic center (21,654). The Holux instruments recorded the most points (44,024) and the Garmin instruments recorded the fewest points (43,685).

The average deviation of all recorded GPS points from the geodetic point coordinates was 12.34 m ± 12.35 m (median 6.34 m). Across all six sites, the Holux RCV-3000 was the most accurate in recording its current static position, with an estimated mean deviation (error) of 11.43 m (95% CI [9.91, 12.96]). The Garmin Forerunner 35 had an average deviation of 12.10 m (95% CI [10.58, 13.62]), while the Qstarz BT-Q1000XT has average deviation of 13.48 m (95% CI [11.96, 15.00]). When averaged across all six sites, there were no significant differences between devices, with the largest difference found between the Holux and Qstarz devices (mean difference = −2.05 m; 95% CI [−4.20, 0.10]; *p* = 0.169).

When combining the Holux and Qstarz data together, the average deviation from the geodetic point was 12.46 m (95% CI [10.82, 14.10]) compared to that of the Garmin, which was 12.10 m (95% CI [9.79, 14.42]). Across all sites, the difference between the Garmin smart watch and the commercial GPS receivers was −0.36 m (95% CI [−3.19, 2.48]; *p* = 0.745).

**Figure 11:**
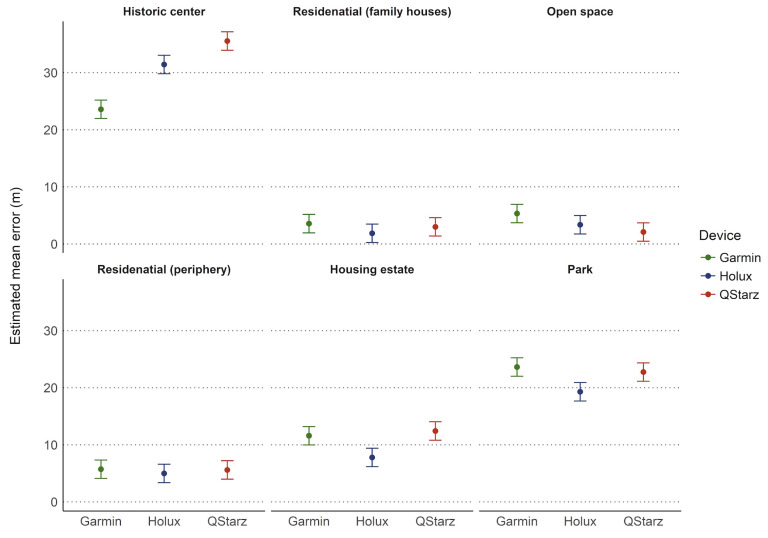
Estimated mean error by type of device across different sites.

When examining the data by site (Figure 11), the Garmin device was significantly more accurate in the historic center compared to the Holux (mean difference = −7.85 m; 95% CI [−11.29, −4.40]; *p* = 0.004) and the Qstarz (mean difference = −11.96 m; 95% CI [−15.40, −8.51]; *p* = 0.001). At this site, the Holux was more accurate than the Qstarz (mean difference = −4.11 m; 95% CI [−7.56, −0.66]; *p* = 0.03). In the housing estate, the Holux had a significantly lower error value compared to both the Qstarz (mean difference = −3.8 m; 95% CI [−7.25, −0.35]; *p* = 0.038) and the Garmin (mean difference = −4.63 m; 95% CI [−8.08, −1.18]; *p* = 0.022). The same trends were also observed for the park site (Table 3). No differences among the devices were observed at any of the other sites.

**Figure 12:**
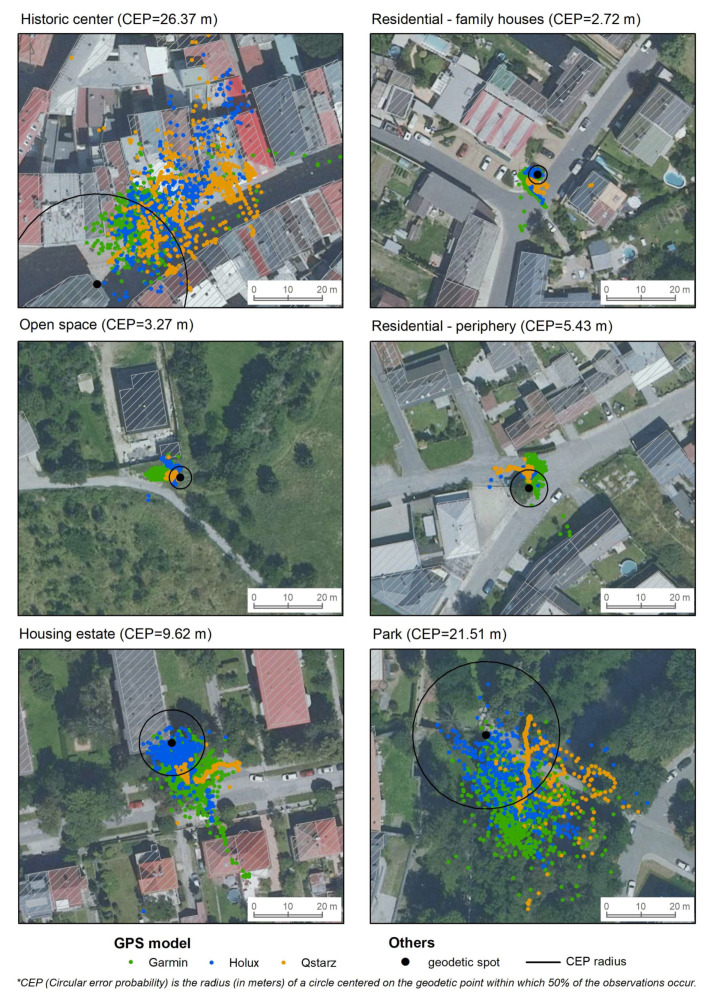
Circular error probability by type of device in different sites.

For the measured points, we also determined the size of the CEP radius, which appropriately reflects both the validity and reliability of the tested instruments and conditions. With respect to this indicator, the GPS instruments located their position most accurately in residential (family houses) (CEP = 2.72 m) and open space areas (CEP = 3.27 m). In both cases, the instruments were not shielded by buildings or vegetation. The least accurate spatial localization was found in the park (CEP = 21.51 m) and the historic center (CEP = 26.37 m). In both cases, the geodetic point was located at the base of a wall, and in the park the GPS signal was shielded by vegetation. As seen in Figure 12, the signal was shielded from the southwest in the historic center and from the northwest in the park. Significant shielding of the GPS signal from the northwest is also observed in the housing estate, where the signal was shielded by a prefabricated house. When averaged across the devices, the pairwise differences in error between each site were all significant (all *p* < 0.001).

## 4. Discussion

This study examined the static accuracy of three different GPS devices (Holux RCV-3000, Qstarz BT-Q1000XT, and Garmin Forerunner 35) across six different sites with varying environmental conditions. There were no differences in accuracy among the three devices when averaged across the six sites. However, there were differences between the devices within three of the six sites, with the Garmin and Holux outperforming the Qstarz device. Considering the fact that the Qstarz BT-Q1000XT instrument is considered by international scientific teams (e.g., IPEN GPS, GPS HRN) as a suitable tool for spatiotemporal localization of motion activity [23,41,42], we also consider the Holux RCV-3000 instrument as a suitable and sufficiently accurate tool. This statement is underlined by the fact that the most accurate of the seven statically tested GPS loggers in the study by Duncan et al. (2013) was the Qstarz BT-Q1000XT. In our study, the Qstarz devices showed the most accurate data only in an environment without any obstruction of the view of the open sky.

The Garmin Forerunner 35 smart watch, which is manufactured specifically for the spatial location of movement activity [43], performs similarly (on average, across all six types of environment) to the Holux and Qstarz BT-Q1000XT in static location accuracy. However, the Garmin smart watch performed better than conventional GPS receivers in the historic city center environment. This also means that the Garmin, like the Qstarz and Holux, could be considered a sufficiently accurate tool. This finding is consistent with the study carried out during the Trollinger Half-Marathon, where an overall mean absolute percentage error of 0.6% was observed in the GNSS-enabled devices, of which the Garmin devices performed the most accurately [38]. The satisfactory location of movement is also confirmed by the fact that none of the distance measurements on the track and field area using the Garmin Forerunner device exceeded a 5% deviation [34].

However, when looking for a suitable instrument for conducting research, accuracy cannot be the only requirement. The internal memory capacity of the Qstarz (8 MB) is higher than that of the Holux (4 MB), which can be a significant advantage in the case of multi-day monitoring [21]. From the experience of the research teams [8], we know that the memory of the Qstarz device will contain recordings for about one week when the recording interval is set to 15 s. The memory capacity of the Garmin smart watch is not specified by the manufacturer, as the user is expected to download stored data more frequently using the Garmin Connect app. Battery life and capacity are significantly higher in the classic GPS receivers. Holux (1050 mAh) and Qstarz (1000 mAh) with Li-ion battery can last for more than 24 h of continuous recording, which is very important for capturing daily travel behaviors [44,45]. In comparison, Garmin introduces a smart watch battery life of 13 h (with GPS on), based on practical verification, rather than within 10 h. When considering the purchase price of the devices, the Holux device (~$60) and Qstarz (~$100) are significantly cheaper than the Garmin smart watch ($200+) as of 2021.

We consider the strength of this study to be the distribution of measurements across six different types of environments. This enables us to reveal differences in accuracy between devices that were not seen in the overall measurements. We also consider the use of two units from each type of device, and the validation of the specifications of the already verified model, to be beneficial.

On the other hand, we are aware of the limitations of collecting data from only one place on Earth and not applying multiple measurements under different atmospheric conditions and at different times. Another limitation is that only static validity was assessed, which may be less relevant than the dynamic accuracy (i.e., comparing GPS trajectories during movement) for physical activity researchers. However, demonstrating the static accuracy is important for identifying the precise locations that someone visits. Further research is needed to investigate the dynamic accuracy of these devices and how their static and dynamic accuracy are related.

## 5. Conclusions

Based on the comparison of the static accuracy of GPS data recording with the verified and expertly recommended Qstarz BT-Q1000XT device, we also consider the Holux RCV-3000 and the Garmin Forerunner 35 smart watch device to be suitable tools for locating physical activity. Researchers must also consider the battery life and storage capacity differences among these devices when determining whether they are suitable for their research studies.

## Figures and Tables

**Table 1:** Specification of the tested GPS devices.

Specification	Qstarz BT-Q1000XT	Holux RCV-3000	Garmin Forerunner 35
Price ($)	100	60	200
Size (mm)	72 × 47 × 20	63 × 41 × 17	36 × 41 × 13
Weight (g)	65	53	37
Chipset	MTK II	MTK MT3329	-
Sensitivity (dBm)	−165	−165	-
Number of channels	66	66	-
Location accuracy [m (%)]	3 (50)	3 (95)	-
Data storage (MB)	8	4	-
Battery (mAh)	1000	1050	-

**Table 2:** Average deviation (m) by device type and the type of environment.

Device Type	Site	Date	Start Time (GTM+1)	N (GPS Points)	Est. Mean Error	95% CI	SD	Median
Garmin	Historic center	30/11/2018	11:27	7036	23.59	[21.98, 25.20]	9.21	22.10
	Residential (family houses)	04/12/2018	15:36	7372	3.56	[1.95, 5.18]	1.92	3.63
	Open space	05/12/2018	14:40	7335	5.33	[3.71, 6.94]	1.36	5.10
	Residential (periphery)	09/12/2018	12:38	7382	5.73	[4.11, 7.34]	2.01	6.05
	Housing estate	09/12/2018	14:28	7386	11.59	[9.98, 13.21]	6.82	10.30
	Park	10/12/2018	16:29	7174	23.63	[22.02, 25.24]	9.40	23.80
Holux	Historic center	30/11/2018	11:27	7366	31.44	[29.82, 33.05]	12.90	27.70
	Residential (family houses)	04/12/2018	15:36	7329	1.87	[0.25, 3.48]	1.09	1.55
	Open space	05/12/2018	14:40	7390	3.36	[1.75, 4.98]	1.37	3.11
	Residential (periphery)	09/12/2018	12:38	7339	4.99	[3.38, 6.61]	0.76	4.99
	Housing estate	09/12/2018	14:28	7400	7.80	[6.18, 9.41]	5.47	5.83
	Park	10/12/2018	16:29	7200	19.30	[17.69, 20.92]	9.59	18.40
Qstarz	Historic center	30/11/2018	11:27	7252	35.55	[33.93, 37.16]	13.5	34.00
	Residential (family houses)	04/12/2018	15:36	7348	3.00	[1.38, 4.61]	0.85	2.90
	Open space	05/12/2018	14:40	7358	2.09	[0.48, 3.71]	0.83	2.12
	Residential (periphery)	09/12/2018	12:38	7345	5.59	[3.98, 7.21]	1.19	5.68
	Housing estate	09/12/2018	14:28	7345	12.42	[10.81, 14.04]	3.24	12.50
	Park	10/12/2018	16:29	7186	22.75	[21.13, 24.36]	7.59	22.10

Note: Estimated mean error (and 95% CI) obtained from linear mixed model.

**Table 3:** Pairwise contrasts between devices across the six sites.

Site	Contrast	Mean Difference (m)	95% CI	p^adj^
Historic center	Garmin-Holux	−7.85	[−11.29, −4.40]	0.004
	Garmin-Qstarz	−11.96	[−15.40, −8.51]	0.001
	Holux-Qstarz	−4.11	[−7.56, −0.66]	0.030
Residential (family houses)	Garmin-Holux	1.70	[−1.75, 5.15]	0.300
	Garmin-Qstarz	0.56	[−2.89, 4.01]	1.000
	Holux-Qstarz	−1.13	[−4.58, 2.32]	0.648
Open space	Garmin-Holux	1.96	[−1.48, 5.41]	0.216
	Garmin-Qstarz	3.24	[−0.21, 6.68]	0.060
	Holux-Qstarz	1.27	[−2.18, 4.72]	0.533
Residential (periphery)	Garmin-Holux	0.73	[−2.72, 4.18]	1.000
	Garmin-Qstarz	0.13	[−3.32, 3.58]	1.000
	Holux-Qstarz	−0.60	[−4.05, 2.85]	1.000
Housing estate	Garmin-Holux	3.80	[0.35, 7.25]	0.038
	Garmin-Qstarz	−0.83	[−4.28, 2.62]	1.000
	Holux-Qstarz	−4.63	[−8.08, −1.18]	0.022
Park	Garmin-Holux	4.33	[0.88, 7.77]	0.026
	Garmin-Qstarz	0.88	[−2.57, 4.33]	0.935
	Holux-Qstarz	−3.45	[−6.89, 0.00]	0.050

Note: Results are differences in model-estimated means obtained from linear mixed models. *p* values are adjusted using the Bonferroni correction.

## Data Availability

The data presented in this study are available on request from the corresponding author. The data are not publicly available due to project privacy.
